# Clinical Relevance and Follow-Up of Incidental CT Imaging Findings for COVID-19 Diagnosis: A Retrospective Analysis

**DOI:** 10.3390/diagnostics15222832

**Published:** 2025-11-08

**Authors:** Marc Marty, Bjarne Kerber, Frederik Abel, Jonas Kroschke, Thomas Frauenfelder, Sabine Franckenberg

**Affiliations:** Diagnostic and Interventional Radiology, University Hospital Zurich, 8091 Zurich, Switzerland; bjarne.kerber@usz.ch (B.K.); frederik.abel@balgrist.ch (F.A.); jonas.kroschke@usz.ch (J.K.); thomas.frauenfelder@usz.ch (T.F.); sabine.franckenberg@usz.ch (S.F.)

**Keywords:** incidental findings, COVID, CT

## Abstract

**Background/Objectives**: The aim of this study was to evaluate the prevalence of incidental findings in thoracic computed tomography (CT) performed because of COVID-19 and their potential impact on patient management. **Methods**: This retrospective analysis included 683 CT scans from 327 patients who underwent CT imaging of the thorax with or without the application of intravenous contrast-agents because of the primary indication of COVID-19. Radiological findings were categorized according to the COVID-19 Pneumonia Imaging Classification by four independent readers. Incidental findings were categorized according to a scale ranging from 0 (no patient impairment) to 3b (severe permanent impairment). **Results**: In the 683 CT-scans, typical COVID-19 findings were present in 273 scans (40.0%), atypical signs in 97 (14.2%), indeterminate findings in 40 (5.9%), and no signs of COVID-19 in 273 (40.0%). Incidental findings were reported in 94 out of 683 cases (13.8%), of which 63 (67.0%) were classified as category 0, 12 (12.8%) as category 1, 9 (9.6%) as category 2a, none (0.0%) as category 2b, 5 (5.3%) as category 3a, and 5 (5.3%) as category 3b. **Conclusions**: CT scans of the thorax for COVID-19 show a small but significant number of incidental findings that require further investigation.

## 1. Introduction

On 30 January 2020, the World Health Organization (WHO) declared COVID-19 a Public Health Emergency of International Concern, and on 11 March 2020, a global pandemic [1]. First identified in Wuhan, China, in December 2019, the infectious disease severely affected public health, economies, and daily life, rapidly overwhelming healthcare systems worldwide.

The most common COVID-19 symptoms are fever, cough, and dyspnea, and gastrointestinal symptoms are less common [2]. SARS-CoV-2 infection can lead to severe complications, such as acute respiratory distress syndrome (ARDS), systemic inflammatory response syndrome (SIRS), and shock [3]. The symptoms vary widely and can mimic other illnesses, thus making clinical presentation alone insufficient for a diagnosis.

Radiology, particularly chest computed tomography (CT), plays a crucial role in diagnosing and monitoring COVID-19-associated pulmonary involvement [4,5,6,7]. Multiple studies have reported that chest CT scans show typical imaging findings in most patients infected with SARS-CoV-2 [8,9,10]. RT-PCR is a reliable standard for diagnosing COVID-19 but has limitations, such as false negatives in the early stages. Chest computed tomography (CT) scans are regularly used to evaluate pneumonia and constitute a helpful tool in diagnosing COVID-19 [6]. A study to evaluate the value of chest CT scans in the diagnosis of COVID-19 found that most patients had an initial positive chest CT scan before or within six days of the initial positive RT-PCR finding [11], whereas other studies found positive diagnoses of COVID-19 with chest CT scans were available 3 days earlier than RT-PCR assays for positive diagnoses of COVID-19 [6]. Another positive aspect is the accessibility of CT, especially in urban areas [12]. Chest computed tomography scans for diagnosing COVID-19 have a high sensitivity (98%) but a low specificity (25%) [6]. The low specificity is mostly due to the overlapping of CT findings shared by diseases caused by viruses from different families, for example, adenovirus, and within the same family, such as SARS-CoV and MERS-CoV [13]. This may also explain why the American College of Radiology (ACR) advises against the use of chest computed tomography (CT) scans as a first-line investigation tool to diagnose COVID-19 [14].

Coronavirus disease 2019 (COVID-19) is associated with increased coagulation activity, which excessively increases the risk of venous thromboembolism (VTE), the most common manifestation being pulmonary embolism (PE), with a risk of approximately one in five hospitalized patients [3]. A prospective cohort study conducted in France reported that 16.7% of 150 patients with COVID-19 developed a PE despite receiving anticoagulant therapy. Diagnosis was confirmed via computed tomography pulmonary angiography (CTPA) with a median detection time of 5.5 days after hospital admission [15]. These findings have been affirmed by different studies with similar results [16,17]. Notably, in patients with confirmed PE, the incidence of deep vein thrombosis (DVT) is significantly lower in individuals with COVID-19 (6.9–13.6%) than in the non-COVID-19 population (45–70%). Moreover, patients with COVID-19 with PE frequently lack the traditional risk factors and comorbidities for PE, unlike non-COVID-19 patients [18]. The effective radiation dose of a chest computed tomography (CT) scan in adults is approximately 3.5 millisieverts (mSv), which corresponds to the amount of natural irradiation received over the course of one year [19].

As with all CT scans, those conducted on COVID-19 patients frequently revealed incidental findings; the observation or discovery of an unexpected and potentially clinically relevant pathology unrelated to the primary indication for imaging and which could not be directly anticipated based on the patient’s clinical presentation or condition [7,20,21,22,23]. As other studies have shown, these incidental findings can have significant clinical implications, presenting opportunities for the early detection of conditions.

This study aimed to (I) assess the prevalence of incidental findings in computed tomography (CT) scans performed for COVID-19 diagnosis, utilizing a large-scale dataset of almost two years (March 2020–December 2022). Additionally, this study sought to (II) evaluate how radiologists and other medical specialties identify, manage, and follow-up on these incidental findings, with a focus on the resulting implications for clinical workflows and patient care.

## 2. Materials and Methods

### 2.1. Study Group

**Figure 1:**
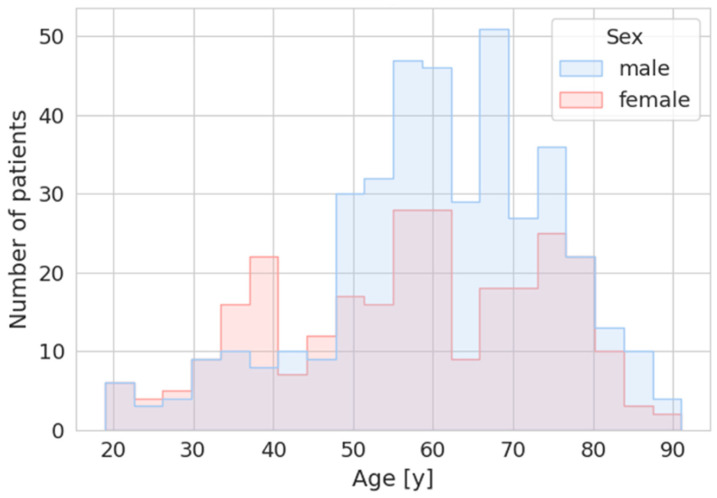
Graphical overview of the distribution of patient numbers and age for males and females in the study group. Age on the *y*-axis in years (y) and number of patients on the *x*-axis.

We analyzed all chest computed tomography scans at the University Hospital of Zurich, Switzerland, that were registered for the clarification of a suspected COVID-19 infection between 16 March 2020, and 31 December 2022. The initial dataset included 1499 chest CT scans, some of which were combined with imaging of other anatomical regions. The incidental findings included in our study were not limited to the thoracic region, all incidental findings were analyzed, regardless of their anatomical location. Exclusion criteria were the absence of general patient consent for data usage or if the primary indication for the examination was not related to COVID-19. Following these exclusion criteria, a total of 683 chest CT scans were included in the final analysis (males *n* = 406, females *n* = 277; mean age males 60.7 ± 14.2 years, mean age females 57.0 ± 16.6 years) (Figure 1).

### 2.2. Imaging

CT scans were performed on three different CT-Scanners (SIEMENS SOMATOM Force, Siemens Healthineers SOMATOM X.cite and SIEMENS SOMATOM Edge Plus, Siemens, Zurich, Switzerland) with different protocols (with and without administration of contrast agent, arterial, arterio-venous or venous phase). A final readout was performed on a RIS/PACS-Workstation by a board-certified radiologist. Retrospective data analysis was performed by four radiology residents under the supervision of a board-certified radiologist. The CT scans were divided among the four resident radiologists, and those with relevant findings were reviewed by the first author.

### 2.3. Radiological Classification of the Imaging Findings

#### 2.3.1. Classification

The radiologic findings were categorized according to the COVID-19 Pneumonia Imaging Classification [24] as follows:

- Typical appearance:
  ▪ Peripheral, bilateral ground-glass opacities (GGO) with or without consolidation or visible intralobular lines (“crazy paving”).
- Indeterminate appearance:
  ▪ Includes one or more of the following features:
  ▪ Multifocal GGO with a rounded morphology, with or without consolidation or visible intralobular lines (“crazy paving”).
  ▪ Reverse halo sign or other patterns suggestive of POP.
  ▪ The absence of typical features, with the presence of multifocal, diffuse, perihilar, or unilateral GGO with or without consolidation lacking a specific distribution. Nonrounded or non-peripheral GGO, or very few small GGO with a non-rounded and non-peripheral distribution, are also included.
- Atypical appearance:
  Defined by the absence of typical or indeterminate features, accompanied by one or more of the following: Isolated or segmental consolidation without GGO, discrete small nodules (centrilobular, “tree-in-bud”), lung cavitation, and smooth interlobular septal thickening with pleural effusion.
- Negative for pneumonia:
  No radiologic features suggestive of pneumonia are present.

#### 2.3.2. Relevance

The incidental findings were evaluated and categorized based on their clinical relevance as follows: 0, no patient impairment; 1, minimal impairment; 2a, mild to moderate temporary impairment; 2b, severe temporary impairment; 3a, mild permanent impairment; and 3b, severe permanent impairment. This grading scheme was chosen to systematically and consistently evaluate the clinical significance of incidental findings, including both the severity and duration of potential patient impairment to ensure consistent interpretation.

### 2.4. Statistics

All analyses were performed using (SPSS, Version 29.0, Chicago, IL, USA). Continuous data are expressed as median with the range or interquartile range (IQR). Categorical data are expressed as numbers and percentages. For the statistical analysis, the chi-square test and the analysis of variance test were used. Continuous data are presented as mean ± standard deviation. Categorical variables were compared using the Chi-square test. Non-parametric tests (Mann–Whitney U test, Kruskal–Wallis test) were applied to assess age differences in the occurrence and relevance of incidental findings.

### 2.5. Ethics and Informed Consent

This study was conducted in accordance with the Declaration of Helsinki and approved by the Institutional Review Board of the Swiss Association of Research Ethics Committees (Basec-Nr. 2019-01676, approval date 24 September 2019). Informed consent was obtained from all subjects involved in the study.

## 3. Results

An overview of the findings and results is presented in Table 1. The anatomical distribution of the incidental findings is detailed in Table 2.

### 3.1. Use of a Contrast Agent

Among the 683 chest CT scans included in the analysis, 323 (47.3%) were initial examinations without any preceding (relevant) CT scan, while 360 (52.7%) were follow-up examinations. Contrast agent administration was utilized in 479 (70.1%) of the CT scans, employing various protocols, including arterial, arteriovenous, or venous phase imaging. A total of 204 scans (29.9%) were performed without the use of a contrast agent.

### 3.2. COVID-Typical Findings

Of the study cohort, 327 patients (47.9%) tested positive for COVID-19, 208 patients (30.5%) had recovered from the infection, 131 patients (19.2%) tested negative for COVID-19, and 17 patients (2.5%) were not tested, with no specific reason provided.

**Figure 2:**
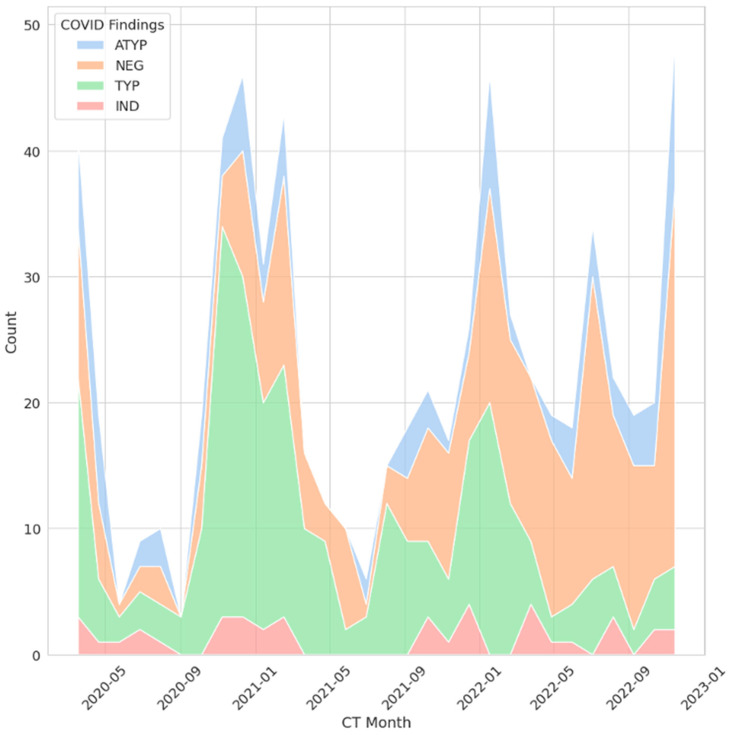
Radiologic COVID-19 findings across all CT examinations. The *x*-axis represents the month and year in which each examination was performed, and the *y*-axis represents the count. ATYP/blue = atypical COVID-findings, NEG/orange = negative for any pneumonia, TYP/green = typical COVID-findings, IND/red = indifferent COVID-findings.

Among the 683 chest CT scans, 273 (40.0%) were negative for COVID-19 pneumonia. Another 273 scans (40.0%) exhibited typical features of COVID-19 pneumonia. Additionally, 97 scans (14.2%) displayed atypical findings, and 40 scans (5.9%) scans were categorized as indeterminate for COVID-19 pneumonia (Figure 2).

### 3.3. Incidental Findings

**Figure 3:**
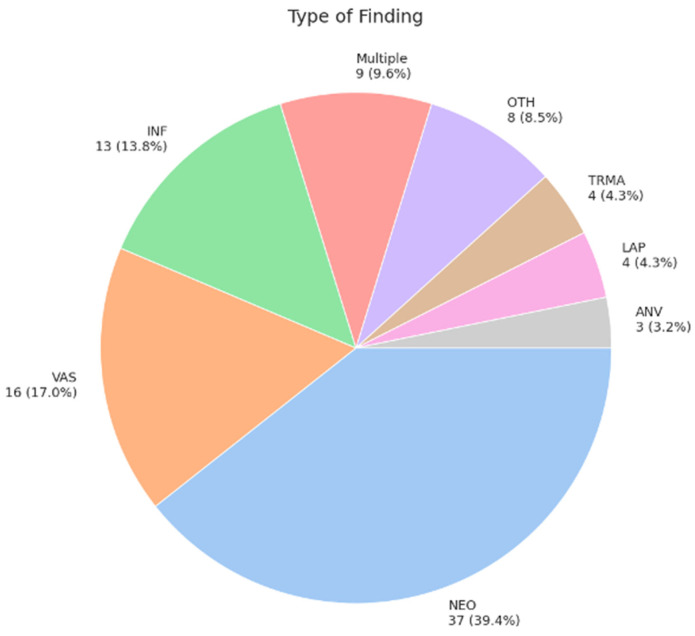
The incidental findings were classified into the following categories: 37 cases (39.4%) were neoplastic (NEO), 16 (17.0%) were vascular (VAS), 13 (13.8%) were inflammatory (INF), 9 (9.6%) involved multiple findings (MULT), 8 (8.5%) were designated as “other” (OTH), for example, an instance with a gastric malrotation, 4 (4.3%) were traumatic (TRMA), 4 (4.3%) were lymphatic (LAP), and 3 (3.2%) represented anatomical variations (ANVs).

**Figure 4:**
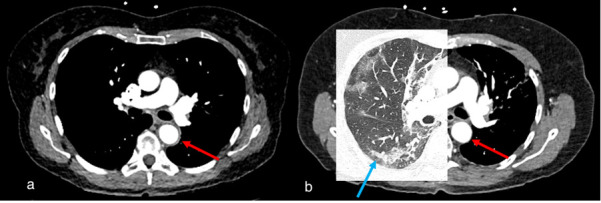
Case 1, a 67-year-old female. (**a**) Initial chest CT to clarify pulmonary embolism or post-COVID-19 findings showed concentric wall thickening of the thoracic aorta (red arrow), indicating large vessel vasculitis. No pulmonary infiltrates or post-COVID-19 findings were observed. (**b**) Follow-up computed tomography 2 months later showed indeterminate appearance of recurrent COVID-19 pneumonia (blue arrow) and regressed arterial wall thickening (red arrow).

**Figure 5:**
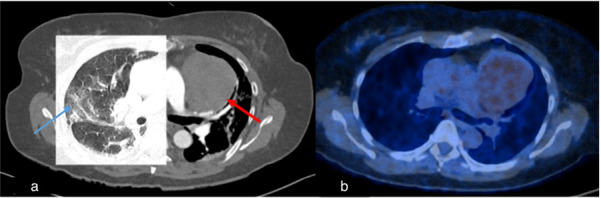
Case 2, 60-year-old female. (**a**) Initial chest CT to clarify pulmonary embolism or COVID-19 findings showed pneumonic infiltrates of indeterminate appearance for COVID-19 (blue arrow) and revealed a large mediastinal mass (red arrow). (**b**) PET/CT demonstrated mild FDG-uptake, making the diagnosis of thymoma more likely than lymphoma.

**Figure 6:**
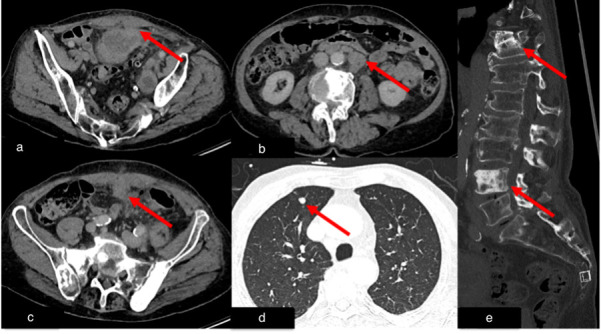
Case 3, 76-year-old male. Initial chest and abdominal CT to investigate potential infections, including COVID-19 findings, revealed no infectious source. However, there was strong suspicion for metastasized carcinoma: (**a**) Wall thickening of a urachal remnant raised concern for urachal carcinoma with infiltration of theabdominal wall (red arrow). (**b**) Enlarged lymph nodes were suspicious for lymph node metastases (red arrow). (**c**) A peritoneal soft tissue mass suggested peritoneal metastasis (red arrow). (**d**) A solid pulmonary nodule in the lung raised concern for lung metastases (red arrow). (**e**) Multiple sclerotic lesions in the spine were indicative of osseous metastasis (red arrow). Histopathological examination following surgical intervention confirmed the diagnosis of metastasized sigmoid carcinoma.

Incidental findings were reported in 94 of 683 cases (13.8%) (Figure 3). Of these, no further evaluation was recommended in 25 cases (26.6%), predominantly due to benign morphological characteristics, although the reasons were unclear in rare instances and will be discussed subsequently. Further radiologic imaging was recommended for 51 patients (54.3%), using CT, MRI, ultrasound (US), or PET/CT (NUC). Biopsy was advised for 3 patients (3.2%), while laboratory parameter evaluation was suggested for 1 patient (1.1%). Fourteen patients (14.9%) were classified as “other,” including cases requiring medication changes or initiation. For instance, a patient with an incidental finding of a left ventricular thrombus had their medication regimen adjusted accordingly. Of the findings, 63 (67.0%) were classified as category 0, 12 (12.8%) as category 1, 9 (9.6%) as category 2a, none (0.0%) as category 2b, 5 (5.3%) as category 3a, and 5 (5.3%) as category 3b. Figure 4, Figure 5 and Figure 6 present typical examples with relevant incidental findings.

### 3.4. Follow-Up or Further Clarification Recommendations

**Figure 7:**
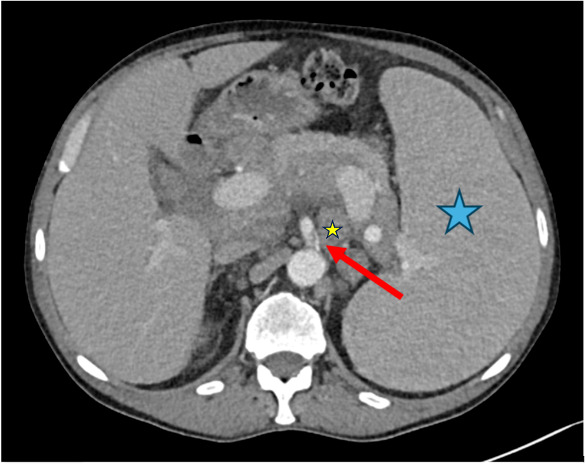
Case 4, 66-year-old male with history of marginal zone lymphoma. A chest CT scan was performed after recovering from COVID-19 to assess post-primary changes in the lung. No pathological lung findings were observed. However, an incidental finding of a short dissection in the superior mesenteric artery was noted (red arrow). Additionally, the spleen (blue asterisk) and retroperitoneal lymph nodes (small yellow asterisk) are enlarged, consistent with the underlying disease.

**Figure 8:**
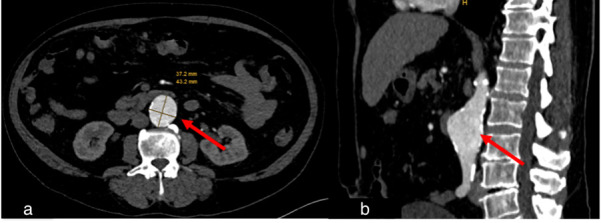
Case 5, 67-year-old male. A chest CT scan was performed to evaluate for pulmonary embolism after recovering from COVID-19. (**a**) Axial view, (**b**) sagittal view. As incidental finding, an abdominal aortic aneurysm was discovered (red arrows).

In 45 cases (47.9%), recommendations for follow-up or further evaluation of incidental findings were not pursued by the clinicians (IGN). Follow-up imaging (FI) was performed in 23 cases (24.5%), 4 cases (4.3%) underwent biopsy, 3 cases (3.2%) underwent surgical intervention, and 1 case (1.1%) underwent laboratory testing for Epstein–Barr virus (EBV) (LAB). Fourteen cases (14.9%) were categorized as “other” (OTH), including instances where patients declined further medical evaluation or passed away before additional diagnostic measures could be undertaken. Figure 7 and Figure 8 show examples of potential suboptimal clinical management despite recommendations in the radiological reports.

### 3.5. Statistical Evaluation of Imaging Findings Among the Subgroups

Incidental findings were observed in 18.4% of female patients (51 out of 277) and 10.6% of male patients (43 out of 406). Women had a significantly higher incidence of incidental findings than men (*p* = 0.005), specifically 73.5% more. There was no significant difference in the detection of incidental findings between non-enhanced and enhanced CTs (*p* = 0.702). No significant association was found between COVID-19 test status and the occurrence of incidental findings (*p* = 0.076). There were significantly more incidental findings in scans with negative COVID-findings (*p* = 0.017).

The ANOVA test results (interval vs. nominal) showed that age had no significant influence (*p* = 0.182).

No significant influence of patient age was found on the presence of incidental findings (Mann–Whitney U test, *p* = 0.169) or on the relevance of findings (Kruskal–Wallis test, *p* = 0.353).

## 4. Discussion

In this retrospective study, we evaluated incidental findings on chest computed tomography (CT) scans performed for COVID-19 diagnosis (I) and their subsequent clinical management, particularly regarding their impact on clinical workflows and patient care (II). Our findings provide valuable insights into the prevalence, categorization, and clinical response to incidental findings in chest CT scans performed during the COVID-19 pandemic.

In this retrospective study, we found a 73.5% higher incidence of incidental findings in women than in men. This might be due to the possibility that women have less reluctance to visit a physician if they feel unwell. Another factor might be that some of the more frequent incidental findings occur more often in women than in men; for example, thyroid cancer is three times more common in women [25]. No significant differences in incidental finding detection rates were observed between non-enhanced and contrast-enhanced CT scans, likely because most incidental findings are visible without contrast. Similarly, neither COVID-19 status nor age significantly influenced incidental findings prevalence. Notably, incidental findings were more frequently detected in CT scans of patients without COVID-19 pneumonia (*n* = 49) than in those with pneumonia (*n* = 44), possibly due to obscuration by lung pathology or “satisfaction of search” bias.

Incidental findings are common in radiology, with reported frequencies of up to 40% [26], often requiring future clinical management [27]. The increasing detection of incidental findings in imaging is no surprise due to the advancements and expanded availability of imaging modalities, especially computed tomography. In urban and well-resourced areas, CT imaging is available around the clock and is often used as a first-line diagnostic tool in emergency and acute care settings. Modern CT-scanners are equipped with features such as dual-energy imaging, iterative reconstruction algorithms, and advanced detector systems, which offer significantly improved resolution and enable a more detailed visualization of anatomical structures. Some state-of-the-art systems can even provide functional assessments like perfusion imaging or material decomposition, for example, iodine maps [7,19,28]. This combination of availability and technological power leads to a higher detection rate of unexpected findings.

A giant cohort study in Ankara, Turkey, investigated the incidence of incidentally identified thyroid nodules on thoracic computed tomography (CT) scans performed for suspicion of COVID-19 pneumonia. The study concluded that incidental thyroid nodules (ITNs) may provide a valuable opportunity for the early detection and treatment of thyroid cancer [21].

Another study was conducted in India to address the frequency and prevalence of incidental findings in COVID-19 screenings. This study highlighted the challenge of balancing the benefits of identifying potential malignancies with the risks of overdiagnosis. It also underscored how the structure of screening programs can influence the nature of incidental findings. For instance, the lack of a widespread breast cancer screening program in India was reflected in the study results, which demonstrated a comparatively high incidence of incidental breast findings, including one confirmed malignancy and nine cases of calcifications/densities, among 100 incidental findings in female patients [22].

A study conducted in Northeast Brazil investigated the prevalence of reported adrenal findings in chest computed tomography scans performed during the COVID-19 pandemic. Although the overall impact of incidental adrenal findings on the healthcare system was minimal, the study highlighted concerns that high workloads may contribute to the underreporting or oversight of incidental findings [23].

A frequently raised concern regarding incidental findings is the potential for over-informing of patients and the impact on healthcare resources, particularly if specialized follow-up care is needed [7,22,23].

However, these studies evaluated rather short time periods with datasets ranging from 2 months [22] to 7 months [21,23].

Incidental findings are expected in CT imaging, and their likelihood increases with age, as the prevalence of undiagnosed pathologies increases. In our cohort, the age distribution peaked in the range of 50–75 years, which aligns with the demographic characteristics of patients typically affected by common conditions, such as pulmonary and thyroid nodules. According to the “National Cancer Institute” of the United States, the median age at cancer diagnosis is 67 years, and most common cancers fall within this range; for example, breast cancer has a median age of 63 years, colorectal cancer has a median age of 66 years, and lung cancer has a median age of 71 years [29].

CT imaging has a relatively high diagnostic accuracy, particularly in identifying incidental findings with sufficient specificity to guide clinical decision-making. In many cases, CT alone provides sufficient detail to characterize incidental lesions and assess their clinical relevance. This is reflected in our study, where 25 cases (26.6%) received recommendations for no further evaluation, direct biopsy (3 patients, 3.2%), laboratory testing (1 patient, 1.1%), or other measures (14 patients, 14.9%) such as medication adjustments. When CT alone is not definitive, a single additional imaging modality is often sufficient to establish a diagnosis [26], a trend also observed in our cohort with 51 patients (54.3%) being advised to undergo further radiologic imaging such as CT, MRI, US, or PET/CT.

The increasing detection of incidental findings has prompted several medical societies, such as the American College of Radiology (ACR), to develop clinical guidelines for their appropriate management [7]. For incidental lung findings, the ACR published the White Paper “Managing Incidental Findings on Thoracic CT: Lung Findings” [30], and the Fleischner Society released the “Guidelines for Management of Incidental Pulmonary Nodules Detected on CT Images” [31]. These guidelines provide evidence-based recommendations for managing unexpected yet common findings, thereby reducing the risk of overlooking significant abnormalities in clinical practice [32].

The data availability for incidental findings is inherently limited, and incidental findings are often not systematically followed up, as our findings suggest, with 47% (45/94) of incidental findings being ignored. This observation is supported by a study conducted by Mabotuwana et al., which demonstrated that the adherence rates are inherently low and vary by modality [33]. Therefore, data on their true clinical impact and long-term outcomes remain scarce.

Our findings suggest that incidental findings frequently lack appropriate follow-up, potentially delaying patient care. While over-reporting may cause unnecessary anxiety [22], radiologists must ensure that referring physicians are effectively informed of clinically significant incidental findings.

And, if a radiology report includes ambiguous language regarding the significance of an incidental finding or lacks clear recommendations for management, it is often beneficial to review the imaging with a clinically experienced radiologist to guide appropriate next steps [34].

A key component in future healthcare systems will be the further development and integration of artificial intelligence (AI) driven clinical decision support systems (CDSSs). These systems can electronically deliver patient-specific recommendations, for example, as reminders or information display. These reminder systems can coordinate guideline-based communication with clinicians and patients. Moreover, reminder systems can proactively identify and engage patients who have not followed management plans and thereby help reducing no-show rates and optimize continuity of care [19,35,36,37].

### Limitations

Limitations of this study are: First, we lacked direct insight into how clinicians acted upon the reported incidental findings, as the analysis relied solely on subsequent medical documentation. Thus, the extent to which incidental findings were addressed remains uncertain. Second, the single-center design may limit the applicability of our findings to other institutions or healthcare facilities. Third, although the radiologists were unaware of study participation due to the retrospective design, a potential reporting bias regarding incidental findings cannot be excluded, as the pandemic-related high workload might have affected reporting behavior. Finally, although inter-reader agreement was achieved, the radiologists who evaluated the CT scans in this study primarily focused on incidental findings rather than the overall accuracy of the reports.

## 5. Conclusions

This study highlights the ongoing challenge of reporting incidental findings and the role of radiologists in guiding clinicians on how to address them. However, the responsibility of follow-up lies outside radiology, contributing to uncertainty regarding incidental finding outcomes. The integration of AI applications could help mitigate some of these challenges by automating aspects of the reporting process and enhancing communication between radiologists, clinicians, and patients [19].

## Figures and Tables

**Table 1:** Overview of the study results. NEG = negative for any type of pneumonia, TYP = typical COVID-findings, ATYP = atypical COVID-findings, IND = indifferent COVID-findings. CT = Computer Tomography, NoFC = no further clarification recommended, OTH = other, US = Ultrasound, MRI = Magnetic Resonance Imaging, NUC = nuclear medicine, BIO = biopsy, LAB = laboratory parameters. IGN = ignored, FI = further imaging done, OTH = other, BIO = biopsy, SUR = surgery, DRU = drugs, LAB = laboratory parameters. Category 0 = “no patient impairment,” 1 = “minimal impairment,” 2a = “mild to moderate temporary impairment,” 2b = “severe temporary impairment,” 3a = “mild permanent impairment,” and 3b = “severe permanent impairment.

Characteristic	Category	Value (*n*, %)
Initial and follow-up imaging	Initial imaging	323 (47.3%)
	Follow-up	360 (52.7%)
The contrast agent used	Yes	479 (70.1%)
	No	204 (29.9%)
COVID Test Result	Positive	327 (47.9%)
	Recovered	208 (30.5%)
	Negative	131 (19.2%)
	Not tested	17 (2.5%)
Radiological CT findings of COVID-19	NEG	273 (40.0%)
	TYP	273 (40.0%)
	ATYP	97 (14.2%)
	IND	40 (5.9%)
Incidental findings	No	589 (86.2%)
	Yes	94 (13.8%)
Recommended Follow-Up Modality	CT	30 (31.9%)
	NoFC	25 (26.6%)
	OTH	14 (14.9%)
	US	9 (9.6%)
	MRI	8 (8.5%)
	NUC	4 (4.3%)
	BIO	3 (3.2%)
	LAB	1 (1.1%)
Follow-Up Consequence	IGN	45 (47.9%)
	FI	23 (24.5%)
	OTH	16 (17.0%)
	BIO	4 (4.3%)
	SUR	3 (3.2%)
	DRU	2 (2.1%)
	LAB	1 (1.1%)
Relevance of the Incidental Findings	0	63 (67.0%)
	1	12 (12.8%)
	2a	9 (9.6%)
	2b	0 (0%)
	3a	5 (5.3%)
	3b	5 (5.3%)

**Table 2:** Overview of the anatomical distribution.

Anatomical Distribution	Specific Location
Thorax (69.3%)	Lung (28.1%) Vascular (16.7%) Osseus (6.1%) Tissue (6.1%) Pleura (4.4%) Lymph nodes (4.4%) Mediastinum (1.8%) Other (1.8%)
Abdomen (30.7%)	Parenchymal (16.7%) Vascular (4.4%) Other (3.5%) Lymph nodes (2.6%) Tissue (2.6%) Osseus (0.9%)

## Data Availability

The data presented in this study are available on request from the corresponding author due to legal restriction.

## References

[1] Cucinotta D., Vanelli M. (2020). WHO Declares COVID-19 a Pandemic. Acta Biomed..

[2] Ochani R., Asad A., Yasmin F., Shaikh S., Khalid H., Batra S., Sohail M.R., Mahmood S.F., Ochani R., Arshad M.H. (2021). COVID-19 pandemic: From origins to outcomes. A comprehensive review of viral pathogenesis, clinical manifestations, diagnostic evaluation, and management. Infez. Med..

[3] Sanidas E., Grassos C., Papadopoulos D., Velliou M., Barbetseas J. (2021). Pulmonary Embolism Prophylaxis in Patients With COVID-19: An Emerging Issue. Heart Lung Circ..

[4] Mossa-Basha M., Meltzer C.C., Kim D.C., Tuite M.J., Kolli K.P., Tan B.S. (2020). Radiology Department Preparedness for COVID-19: Radiology Scientific Expert Review Panel. Radiology.

[5] Darwish H.S., Habash M.Y., Habash W.Y. (2021). Chest computed tomography imaging features in patients with coronavirus disease 2019 (COVID-19). J. Int. Med. Res..

[6] Alsharif W., Qurashi A. (2021). Effectiveness of COVID-19 diagnosis and management tools: A review. Radiography.

[7] Battegay E. (2017). Differenzialdiagnose Innerer Krankheiten: Vom Symptom zur Diagnose.

[8] Zhao W., Zhong Z., Xie X., Yu Q., Liu J. (2020). Relation Between Chest CT Findings and Clinical Conditions of Coronavirus Disease (COVID-19) Pneumonia: A Multicenter Study. Am. J. Roentgenol..

[9] Salehi S., Abedi A., Balakrishnan S., Gholamrezanezhad A. (2020). Coronavirus Disease 2019 (COVID-19): A Systematic Review of Imaging Findings in 919 Patients. Am. J. Roentgenol..

[10] Caruso D., Zerunian M., Polici M., Pucciarelli F., Polidori T., Rucci C., Guido G., Bracci B., De Dominicis C., Laghi A. (2020). Chest CT Features of COVID-19 in Rome, Italy. Radiology.

[11] Ai T., Yang Z., Hou H., Zhan C., Chen C., Lv W., Tao Q., Sun Z., Xia L. (2020). Correlation of Chest CT and RT-PCR Testing for Coronavirus Disease 2019 (COVID-19) in China: A Report of 1014 Cases. Radiology.

[12] Tailor T.D., Tong B.C., Gao J., Choudhury K.R., Rubin G.D. (2019). A Geospatial Analysis of Factors Affecting Access to CT Facilities: Implications for Lung Cancer Screening. J. Am. Coll. Radiol..

[13] Li Y., Xia L. (2020). Coronavirus Disease 2019 (COVID-19): Role of Chest CT in Diagnosis and Management. Am. J. Roentgenol..

[14] ACR (American College of Radiology) ACR Recommendations for the Use of Chest Radiography and Computed Tomography (CT) for Suspected COVID-19 Infection. https://www.acr.org/Advocacy/Position-Statements/Recommendations-for-Chest-Radiography-and-CT-for-Suspected-COVID19-Infection.

[15] Helms J., Tacquard C., Severac F., Leonard-Lorant I., Ohana M., Delabranche X., Merdji H., Clere-Jehl R., Schenck M., Gandet F.F. (2020). High risk of thrombosis in patients with severe SARS-CoV-2 infection: A multicenter prospective cohort study. Intensive Care Med..

[16] Klok F.A., Kruip M.J.H.A., van der Meer N.J.M., Arbous M.S., Gommers D.A.M.P.J., Kant K.M., Kaptein F.H.J., van Paassen J., Stals M.A.M., Huisman M.V. (2020). Incidence of thrombotic complications in critically ill ICU patients with COVID-19. Thromb. Res..

[17] Llitjos J.-F., Leclerc M., Chochois C., Monsallier J.-M., Ramakers M., Auvray M., Merouani K. (2020). High incidence of venous thromboembolic events in anticoagulated severe COVID-19 patients. J. Thromb. Haemost..

[18] Trunz L.M., Lee P., Lange S.M., Pomeranz C.L., Needleman L., Ford R.W., Karambelkar A., Sundaram B. (2021). Imaging approach to COVID-19 associated pulmonary embolism. Int. J. Clin. Pract..

[19] Huisman M., Kotter E., van Ooijen P.M.A., Ranschaert E.R., Antonoli T.A.D., Rockenbach M.A.B.C., Silva V.C.E., Koltsakis E., Yilmaz P. (2023). ESR Modern Radiology eBook.

[20] Frank L., Quint L.E. (2012). Chest CT incidentalomas: Thyroid lesions, enlarged mediastinal lymph nodes, and lung nodules. Cancer Imaging.

[21] Helvacı B.C., Ozdemir D., Turan K., Keskin C., İmGa N.N., Dirikoc A., Topaloglu O., Ersoy R., Cakir B. (2024). Incidental thyroid nodules on COVID-19-related thoracic tomography scans: A giant cohort. Hormones.

[22] Valluri S., Lakshmi H.N., Sunkavalli C. (2023). Incidental Findings in CT Scans on Screening for COVID-19. Indian J. Surg. Oncol..

[23] de Magalhães L.J.T., Rocha V.G., de Almeida T.C., de Albuquerque E.V. (2023). Prevalence of reported incidental adrenal findings in chest computerized tomography scans performed during the COVID-19 pandemic in a single center in Northeast Brazil. Arch. Endocrinol. Metab..

[24] Simpson S., Kay F.U., Abbara S., Bhalla S., Chung J.H., Chung M., Henry T.S., Kanne J.P., Kligerman S., Ko J.P. (2020). Radiological Society of North America Expert Consensus Statement on Reporting Chest CT Findings Related to COVID-19. Endorsed by the Society of Thoracic Radiology, the American College of Radiology, and RSNA—Secondary Publication. J. Thorac. Imaging.

[25] Remer L.F., Lee C.I., Picado O., Lew J.I. (2022). Sex Differences in Papillary Thyroid Cancer. J. Surg. Res..

[26] Wünnemann F., Rehnitz C., Weber M.A. (2017). Incidental findings in musculoskeletal radiology. Radiologe.

[27] Leitman B.S. (2018). Comment on the Avoidance of Reporting Incidental Findings. J. Am. Coll. Radiol..

[28] McCollough C.H., Leng S., Yu L., Fletcher J.G. (2015). Dual- and Multi-Energy CT: Principles, Technical Approaches, and Clinical Applications. Radiology.

[29] NCI Age and Cancer Risk. National Cancer Institute. https://www.cancer.gov/about-cancer/causes-prevention/risk/age#:~:text=Age%20and%20Cancer%20Risk,-Advancing%20age%20is&text=The%20incidence%20rates%20for%20cancer,groups%2060%20years%20and%20older.

[30] Munden R.F., Black W.C., Hartman T.E., MacMahon H., Ko J.P., Dyer D.S., Naidich D., Rossi S.E., McAdams H.P., Goodman E.M. (2021). Managing Incidental Findings on Thoracic CT: Lung Findings. A White Paper of the ACR Incidental Findings Committee. J. Am. Coll. Radiol..

[31] MacMahon H., Naidich D.P., Goo J.M., Lee K.S., Leung A.N.C., Mayo J.R., Mehta A.C., Ohno Y., Powell C.A., Prokop M. (2017). Guidelines for Management of Incidental Pulmonary Nodules Detected on CT Images: From the Fleischner Society 2017. Radiology.

[32] Hillman B.J. (2017). Incidental. J. Am. Coll. Radiol..

[33] Mabotuwana T., Hombal V., Dalal S., Hall C.S., Gunn M. (2018). Determining Adherence to Follow-up Imaging Recommendations. J. Am. Coll. Radiol..

[34] Brett A.S. (2024). Incidental Findings in Chest CT. NEJM Journal Watch (General Medicine). https://www.jwatch.org/na57940/2024/09/17/incidental-findings-chest-ct.

[35] Sutton R.T., Pincock D., Baumgart D.C., Sadowski D.C., Fedorak R.N., Kroeker K.I. (2020). An overview of clinical decision support systems: Benefits, risks, and strategies for success. npj Digit. Med..

[36] Langenbach M.C., Foldyna B., Hadzic I., Langenbach I.L., Raghu V.K., Lu M.T., Neilan T.G., Heemelaar J.C. (2025). Automated anonymization of radiology reports: Comparison of publicly available natural language processing and large language models. Eur. Radiol..

[37] Sangal R.B., Sharifi M., Rhodes D., Melnick E.R. (2023). Clinical Decision Support: Moving Beyond Interruptive “Pop-up” Alerts. Mayo Clin. Proc..

